# Phase 1 Dose Escalation Study of the Allosteric AKT Inhibitor BAY 1125976 in Advanced Solid Cancer—Lack of Association between Activating AKT Mutation and AKT Inhibition-Derived Efficacy

**DOI:** 10.3390/cancers11121987

**Published:** 2019-12-10

**Authors:** Andreas Schneeweiss, Dagmar Hess, Markus Joerger, Andrea Varga, Stacy Moulder, Apostolia M. Tsimberidou, Cynthia Ma, Sara A. Hurvitz, Christine Rentzsch, Marion Rudolph, Silke Thiele, Oliver Boix, Gary Wilkinson, Eleni Lagkadinou, Matthias Ocker

**Affiliations:** 1 National Center for Tumor Diseases, University Hospital Heidelberg and German Cancer Research Center, 69120 Heidelberg, Germany; 2 Kantonsspital St. Gallen, 9001 St. Gallen, Switzerland; dagmar.hess@kssg.ch (D.H.); markus.joerger@kssg.ch (M.J.); 3 Institut Gustave Roussy, 94800 Villejuif, France; andrea.varga@gustaveroussy.fr; 4 MD Anderson Cancer Center, Houston, TX 77030, USA; smoulder@mdanderson.org (S.M.); atsimber@mdanderson.org (A.M.T.); 5 Department of Medicine, Washington University School of Medicine, St. Louis, MO 63110, USA; cma@dom.wustl.edu; 6 David Geffen School of Medicine, University of California Los Angeles, Los Angeles, CA 90095, USA; shurvitz@mednet.ucla.edu; 7 Bayer AG, 13353 Berlin, Germany; christine.rentzsch@bayer.com (C.R.); marion.rudolph@bayer.com (M.R.); silke.thiele@bayer.com (S.T.); oliver.boix@bayer.com (O.B.); gary.wilkinson@bayer.com (G.W.); eleni.lagkadinou@bayer.com (E.L.); matthias.ocker@bayer.com (M.O.)

**Keywords:** AKT inhibitor, biomarker, breast cancer, pharmacokinetics, phase 1

## Abstract

This open-label, phase I first-in-human study (NCT01915576) of BAY 1125976, a highly specific and potent allosteric inhibitor of AKT1/2, aimed to evaluate the safety, pharmacokinetics, and maximum tolerated dose of BAY 1125976 in patients with advanced solid tumors. Oral dose escalation was investigated with a continuous once daily (QD) treatment (21 days/cycle) and a twice daily (BID) schedule. A dose expansion in 28 patients with hormone receptor-positive metastatic breast cancer, including nine patients harboring the *AKT1^E17K^* mutation, was performed at the recommended phase 2 dose (R2D) of 60 mg BID. Dose-limiting toxicities (Grades 3–4) were increased in transaminases, γ-glutamyltransferase (γ-GT), and alkaline phosphatase in four patients in both schedules and stomach pain in one patient. Of the 78 patients enrolled, one patient had a partial response, 30 had stable disease, and 38 had progressive disease. The clinical benefit rate was 27.9% among 43 patients treated at the R2D. *AKT1^E17K^* mutation status was not associated with tumor response. Genetic analyses revealed additional mutations that could promote tumor cell growth despite the inhibition of AKT1/2. BAY 1125976 was well tolerated and inhibited AKT1/2 signaling but did not lead to radiologic or clinical tumor responses. Thus, the refinement of a selection of biomarkers for AKT inhibitors is needed to improve their monotherapy activity.

## 1. Introduction

Precision medicine aims to identify patients based on distinct molecular profiles to provide optimal and more effective treatments with reduced side effects. Breast cancer remains the most common malignant disease in women worldwide [1], and despite improvements in outcome for early stages of hormone receptor-positive (HR+) and HER2 negative breast cancer, advanced stages of the disease still represent a high medical burden [2].

Alterations in the signaling pathways that regulate cellular survival and growth are a hallmark of cancer [3]. Among these pathways, the PI3K/AKT/mTOR signaling cascade has been described as frequently activated in different solid tumors, including up to 60% in breast cancers according to a recent The Cancer Genome Atlas (TCGA) analysis [4]. The substitution of glutamic acid to lysine by a somatic point mutation (E17K) renders the serine/threonine kinase AKT1 independent of upstream PI3K signaling and leads to constitutive activation [5,6]. Within comprehensive molecular profiling studies of breast cancer tissue, the *AKT1^E17K^* mutation was identified as a probable oncogenic driver in patients, indicating that the inhibition of AKT1 presents a novel and specific drug target in this disease [5,6]. However, as the prevalence of the *AKT1^E17K^* mutation was considered low (6.3%), a patient selection strategy was necessary.

Several (pan-)AKT inhibitors have been developed recently [7]. These compounds are either ATP-competitive (e.g., AZD5363 [8]) or allosteric (e.g., MK-2206 [9]) inhibitors and were investigated in various indications. BAY 1125976 is an oral, small-molecule allosteric inhibitor of AKT1/2 with high selectivity. It inhibits the proliferation of cells with PI3K/AKT/mTOR pathway alterations at submicromolar IC_50_ values and showed its highest activity in luminal breast cancer cell lines. BAY 1125976 exhibited in vivo antitumor activity in preclinical breast cancer models after oral application [10]. Furthermore, a potent inhibition of the downstream signaling cascade was demonstrated by reduced levels of p-AKT, p-PRAS40, p-S6RP, or p-70S6K, leading to antitumor efficacy in *AKT1^E17K^*-mutated prostate and anal cancer models [10].

Here, we report the first-in-human phase I study that evaluated the safety, tolerability, pharmacokinetics, and maximum tolerated dose (MTD) of BAY 1125976 in patients with advanced solid tumors (NCT01915576). In an expansion phase, an enrichment strategy to identify patients with *AKT1^E17K^*-mutated tumors was implemented. In addition, we will discuss options for further precision medicine approaches in similar patient populations.

## 2. Results

### 2.1. Baseline Patient Demographics

**Figure 1:**
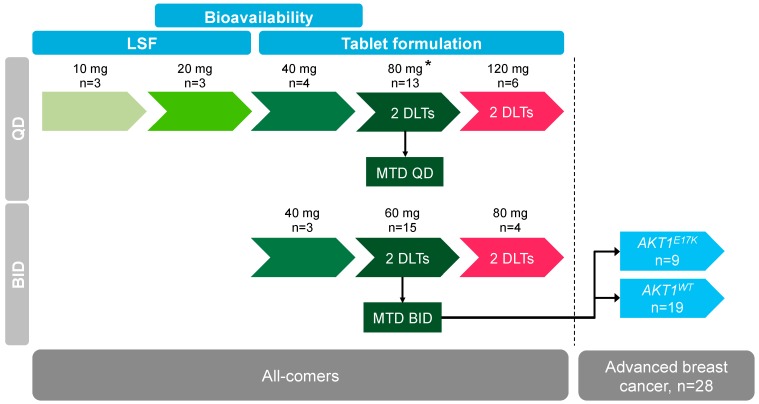
Study outline and patient disposition during dose escalation steps and for the breast cancer expansion cohort at 60 mg BID BAY 1125976.

A total of 79 patients were enrolled in this study (Table 1, Table S1). Twenty-nine patients received the continuous once daily (QD) dose escalation schedule, while 22 patients were part of the twice daily (BID) dose escalation. The breast cancer expansion cohort using 60 mg BID consisted of 28 patients and was discontinued after the enrollment of eight patients with *AKT1^E17K^* mutation (Figure 1). Overall, 61 patients (77.2%) were female. The mean age of all the patients was 56.7 years (range 31–82 years). Except for one patient in the 80 mg QD dose escalation and one patient in the 80 mg BID dose escalation, all patients had a baseline Eastern Cooperative Oncology Group (ECOG) performance status of 0 (59.5%) or 1 (38.0%). Only one patient (40 mg BID cohort) did not receive any prior systemic anticancer therapy. Sixty patients (75.9%) had prior radiotherapy. The baseline characteristics for patients with *AKT1^E17K^* mutations were comparable to the whole study population.

### 2.2. Dose Escalation and Maximum Tolerated Dose

During the initial QD dose escalation, no dose-limiting toxicity was observed until the 120 mg QD cohort. Here, two patients experienced Grade 3 or Grade 4 liver enzyme elevation of aspartate animotransferase (AST), alanine animotransferase (ALT), γ-glutamyltransferase (γ-GT), and one patient experienced Grade 3 elevation of alkaline phosphatase (AP). Based on PK and modeling data, a re-escalation using a BID schedule was initiated at 40 mg BID with the intent of maintaining target engagement whilst reducing C_max_ under the hypothesis that higher C_max_ may be linked to observed events. Two patients at the 80 mg BID dose level experienced Grade 3 liver enzyme elevation (AST, ALT), with one of these patients also showing Grade 3 hyperglycemia. Dose was then de-escalated to 60 mg BID and two patients experienced Grade 3 liver enzyme elevation (AST, ALT, γ-GT), too. The MTD estimate based on posterior dose-limiting toxicity (DLT) rates of the Bayesian dose response analysis was 81.1 mg for the QD schedule (with a coefficient of variation of 25.5%) and 65.1 mg for the BID schedule (with a coefficient of variation of 38.1%), respectively. Therefore, the MTD and recommended dose for the expansion phase was selected as 60 mg BID.

### 2.3. Safety

During the study, 77 (97.5%) patients reported at least one treatment-emergent adverse event (TEAE), drug-related TEAEs were reported by 69 (87.3%) patients, and nine (11.4%) patients had TEAEs related to procedures required by the protocol. Eighteen (22.8%) patients had TEAEs that were Common Terminology Criteria for Adverse Events (CTCAE) Grade 1 or Grade 2, and 52 (65.8%) patients had TEAEs that were CTCAE Grade 3 or Grade 4, with overall only one patient in the 120 mg QD cohort experiencing a Grade 4 increase of γ-GT. Most of the common Grade 3 drug-related TEAEs were increased ALT in 17 patients (21.5%), increased AST in 20 patients (25.3%), and increased AP in 27 patients (34.2%) (Table 2). Interestingly, only one patient experienced an on-target Grade 3 hyperglycemia. Six patients died during the treatment period or within the 30 days safety follow-up window. All deaths were due to disease progression and considered not related to BAY 1125976. Serious adverse events (SAEs) were reported in 33 patients (41.8%). Six SAEs were considered to be drug-related. Forty-five (57.0%) patients had TEAEs leading to dose modification, and 22 (27.8%) patients had TEAEs leading to permanent discontinuation of the study drug.

### 2.4. Pharmacokinetic Evaluation

**Figure 2:**
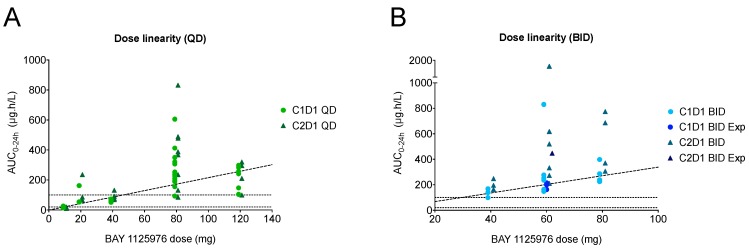
Single and multiple dose area under the concentration time curve (AUC_0-24h_) of BAY 1125976 in the QD (**A**) and the BID (**B**) schedule, including patients at the 60 mg BID expansion cohort, showing dose linearity for both schedules. C, cycle; D, day; Exp, expansion cohort.

BAY 1125976 was rapidly absorbed following continuous QD or BID oral administration of the liquid service formulation (LSF) or the tablet formulation. Median time to reach t_max_ was in the range of 0.5 to 3 h. Exposure was observed to increase with dose up to the 80 mg dose level in both schedules (Figure 2, Table S2). The relative bioavailability assessment of BAY 1125976 (tablet versus LSF) at the 20 mg QD dose level and at the 40 mg QD dose level showed comparable C_max_ and area under the concentration time curve (AUC) values for both formulations (Table S3, Figure S1). The administration of BAY 1125976 at the 80 mg QD dose level after a high-fat, high-calorie meal resulted in a reduction of C_max_ by 27% and an increase of AUC by 16% compared to administration under fasted conditions (Table S4, Figure S1). There was a delay in absorption when the tablet was taken with food (median t_max_ was 1 h and 4.1 h under fasted and fed conditions, respectively). Due to the small sample size in the food effect cohort (*n* = 3), the data should be interpreted with caution.

### 2.5. Pharmacodynamic Biomarkers

**Figure 3:**
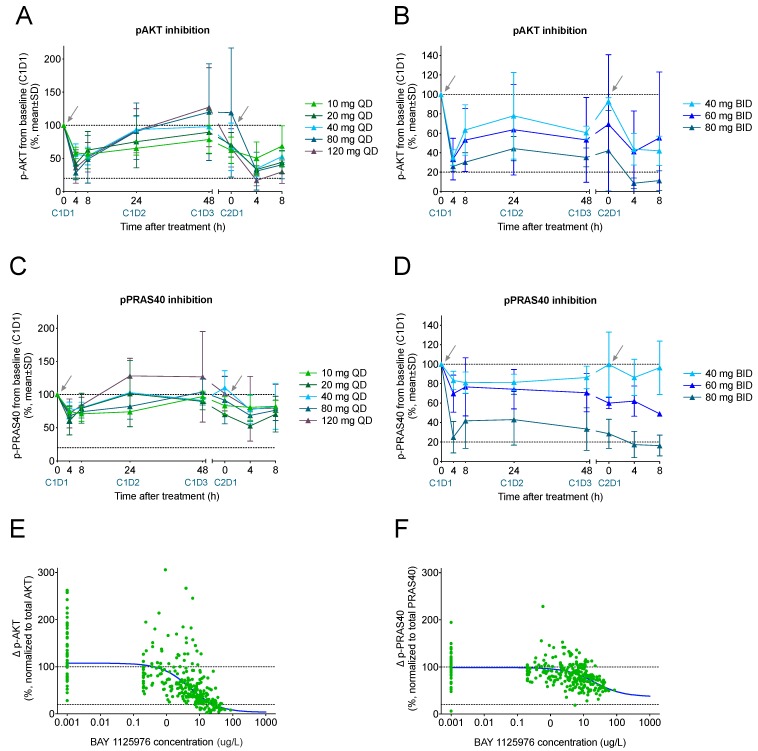
Inhibition of phosphorylation of AKT (**A**,**B**) and PRAS40 (**C**,**D**) as pharmacodynamic biomarkers in platelet-rich plasma (PRP) from patients in the QD (left column) and BID (right column) dose escalation parts in the BAY 1125976 Phase 1 study. PK/PD analysis on the suppression of p-AKT (**E**) and p-PRAS40 (**F**) from thrombin receptor-activating peptide (TRAP)-stimulated platelets across dose intervals relative to baseline (screening, C1D1 predose or C1D-3 predose). Values are normalized to total AKT and total PRAS40, respectively. Vertical dotted line represents IC_90_ for p-AKT based on clinical pharmacokinetic/pharmacodynamic (PK/PD) modeling. Arrows indicate when treatments were started. C, cycle; D, day. PD: progressive disease.

To determine the degree of inhibition of the AKT signaling pathway by BAY 1125976, inhibition of the phosphorylation of AKT and of its downstream target PRAS40 was determined in platelet-enriched plasma samples after thrombin receptor-activating peptide (TRAP) stimulation (Figure 3). A transient maximum inhibition of AKT phosphorylation was observed at 4 h post-treatment in both dosing schedules (Figure 3A,B). While a decrease of p-AKT quickly returned to baseline in the QD schedule, a sustained inhibition was observed for patients receiving BAY 1125976 in the BID schedule. Similar data were obtained for the inhibition of phosphorylation of PRAS40 (Figure 3C,D). Overall, a clear dose and exposure dependency of both p-AKT and p-PRAS40 inhibition was seen. PK/PD (pharmacokinetic/pharmacodynamic) data demonstrated a suppression of more than 70% of p-AKT and more than 30% of p-PRAS40 at the MTD dose level with an IC_50_ of approximately 5 µg/L for p-AKT (Figure 3E,F). Although mandatory for patients in the expansion cohort, due to early disease progression, some planned biopsies were not taken, and only 10 treatment biopsies were collected on cycle 2, day 1. Of these, eight did not meet the prespecified quality criteria (e.g., relative tumor content). Therefore, no further analyses of tissue-specific pharmacodynamic biomarkers were performed due to the low number of evaluable cases.

### 2.6. AKT1^E17K^ Status and Mutational Profiling

All patients with tumor *AKT1^E17K^* mutation enrolled in the study were identified based on prior investigations at the different sites. All mutations were confirmed retrospectively using the Therascreen assay. The de novo screening of a further 18 patients was not able to identify the *AKT1^E17K^* mutation, which was in line with the expected low prevalence of the mutation.

**Figure 4:**
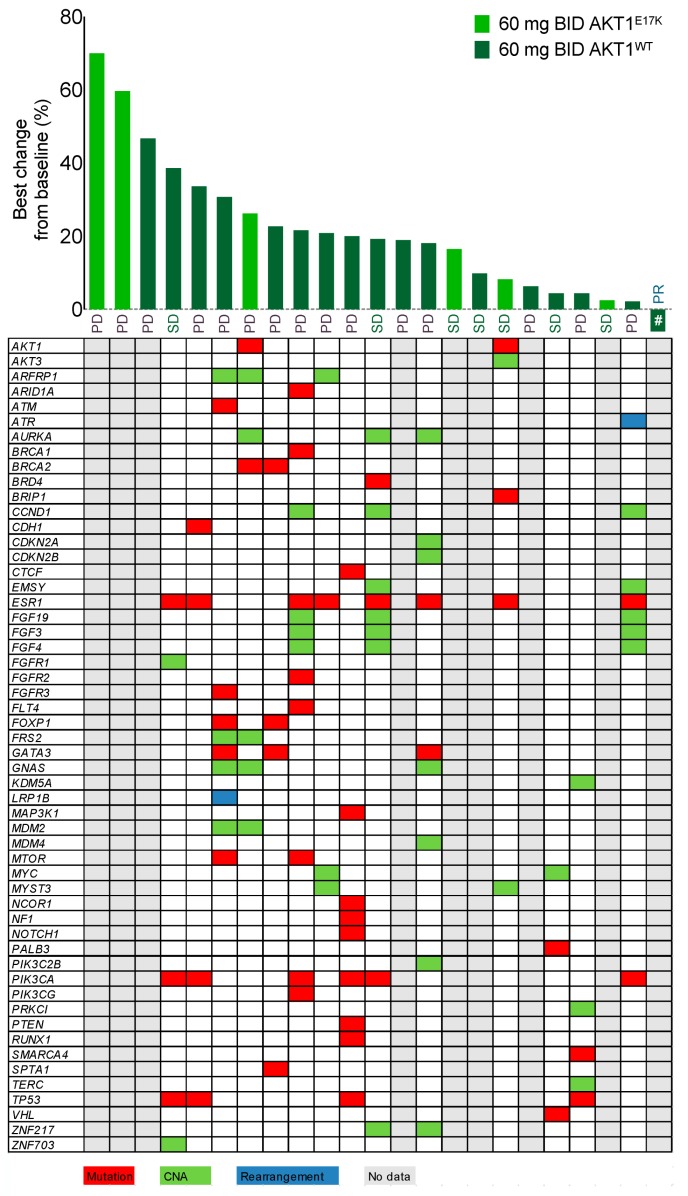
Waterfall plot showing the best change (%) from baseline and response evaluation according to RECIST v1.1 for patients from the 60 mg BID breast cancer expansion cohort (*n* = 28) in the BAY 1125976 phase 1 study. The *AKT1^E17K^* mutation status for each patient was detected from tumor specimen using the Therascreen assay or based on pre-existing data determined at the investigational site. The table depicts genetic aberrations with known or likely oncogenic properties in the target population as found by next-generation sequencing (NGS) in tumor samples. # indicates the patient who had partial response (−36.5% from baseline). PR, partial response; SD, stable disease; PD, progressive disease. Color indications: Red, mutation; Green, copy number alteration (CNA); Blue, rearrangement; Gray, no data available (failed analysis, low/no tumor content in sample or missing sample).

Next-generation sequencing revealed additional oncogenic mutations in the analyzed samples from the 60 mg BID breast cancer expansion cohort (*n* = 28). Only samples from patients with efficacy evaluation by Response Evaluation Criteria in Solid Tumors (RECIST) were analyzed (*n* = 23), and results were obtained for 14 patients. Other samples failed quality control or had too low tumor content. The *AKT1^E17K^* mutation was not mutually exclusive to other known or potential oncogenic drivers. The alterations identified in our study population also included other PI3K pathway members, e.g., *PIK3CA* mutation, *PTEN*, or *RICTOR*, as well as e.g., *TP53, BRCA1/2, KRAS, MYC*, or *FGFR1* and *FGFR2*. Copy number alterations (CNAs) were observed in e.g., cell cycle control genes (*CDKN2A/B, CDK6*), fibroblast growth factor (FGF) signaling (*FGF3, FGF4, FGF19*), *MDM2*, or *AURKA* (Figure 4, Figure S2). Overall, an average (min–max) of seven (3–12) mutations or CNAs with known or likely oncogenic properties was detected per patient. Due to the low number of patients, no statistical differences could be detected between patients with wild-type (WT) and *AKT1^E17K^*-mutated tumors. Not surprisingly for our target population, alterations were most frequently found in *ESR1* (estrogen receptor α), *PIK3CA,* and *TP53*.

### 2.7. Tumor Response Evaluation

Seventy-eight patients were evaluable for tumor response. The average number of prior lines of therapy was 5.8 (range 0–14). No patient had a complete response, and one patient (1.3%) treated with 60 mg BID in the expansion cohort had a partial response as best response (−36.5% best change in target lesions from baseline). Of the other overall 77 patients, 30 (38.5%) patients had stable disease, 38 (48.7%) patients had progressive disease, and data were missing for nine patients. At the 60 mg BID expansion cohort (Figure 4), the above-mentioned one patient (3.6%) had a partial response, seven patients (25.0%) had stable disease, and 15 patients (53.6%) had progressive disease (no data available for five (17.9%) patients). Of the nine patients recruited into the study with the *AKT1^E17K^* mutation, three (33.3%) patients had stable disease, three (33.3%) patients had progressive disease, and data were missing for the other three patients. The overall median (95% confidence interval) time to disease progression for the 78 evaluable patients was 38 (36, 43) days and 41 (22, 80) days for the nine patients with the *AKT1^E17K^* mutation.

## 3. Discussion

Alterations of the PI3K/AKT/mTOR pathway belong to the most commonly found molecular changes in human cancers, including breast cancer [4]. Early studies using inhibitors of PI3K (phosphoinositide 3-kinase) (e.g., copanlisib [11], buparlisib [12] or alpelisib [13]) or downstream mTOR (mammalian target of rapamycin) signaling complexes (e.g., temsirolimus [14,15] or everolimus [16,17]) in monotherapy indicate some clinical benefit in patients with advanced breast cancer. Therefore, targeting the intermediate signaling step via the inhibition of AKT could be an additional way to inhibit tumor cell growth, especially in cancers unresponsive to PI3K inhibitors. Activating mutations of the *AKT1* gene have been described in various solid tumors, including breast cancer [5]. The *AKT1^E17K^* point mutation leads to the constitutive activation of the kinase in 6.3% of human breast cancer [6]. Although this prevalence is rather low, novel sequencing and detection technologies, including next-generation sequencing and respective biobanking approaches at clinical sites, identified a total of nine patients with the *AKT1^E17K^* mutation for this phase 1 study. The further penetration of such technologies into routine diagnostics will make even more rare alterations (e.g., *NTRK* gene fusions) attractive targets for future drug development scenarios [18].

BAY 1125976 is a highly potent and oral specific allosteric inhibitor of AKT1/2 and demonstrated antitumor activity in several preclinical cancer models, including breast cancer [10]. Therefore, this phase 1 first-in-human study was initiated to determine the safety, tolerability, pharmacokinetics, and MTD of BAY 1125976 in patients with advanced cancer.

The study started using a QD dose escalation scheme, and the occurrence of dose-limiting liver toxicity as evidenced by the Grade 3 and Grade 4 elevation of transaminases, AP, or γ-GT at 120 mg led to the identification of 80 mg as the MTD in the QD schedule. The observed hepatotoxicity is considered to be a direct effect of prolonged inhibition of the PI3K/AKT/mTOR pathway in hepatocytes where it regulates glucose homeostasis and other key metabolic pathways [19,20]. Preclinically, BAY 1125976 strongly reduced levels of p-AKT and p-PRAS40 after a single oral dose in tumor xenografts [10]. We found a dose-dependent inhibition of both pharmacodynamic biomarkers with maximum inhibition being detectable at 4 h post-dosing and a rapid return to baseline suggesting a rapid direct effect link between AKT inhibition and biomarker modulation. This is in line with our preclinical data that identified the inhibition of p-AKT and p-PRAS40 as pharmacodynamic biomarkers [10]. PK/PD data demonstrated that the QD administration, even at the MTD, was not able to achieve sustained coverage above the in vitro IC_50_ of p-AKT or p-PRAS40. Pharmacokinetic simulations of potential other schedules predicted continuous IC50 coverage using a BID schedule. Therefore, this schedule was initiated as an alternative dose escalation cohort starting at 40 mg BID. In this cohort, three escalation steps up to 80 mg BID were performed, and Grade 3 liver toxicities were again identified as dose-limiting. In line with the predictions, an assessment of p-AKT and p-PRAS40 confirmed sustained inhibition with this schedule. The MTD was determined as 60 mg BID. Then, this dose level was used for further expansion in the breast cancer cohort. We observed uniform dose-limiting toxicities, i.e., liver enzyme elevations and hyperglycemia, which are in line with the expected mode of action of AKT inhibitors [19,20].

Although ATP-competitive AKT inhibitors such as AZD5363 or GSK690693 have shown encouraging preclinical results, this compound class shows significant clinical toxicity (hyperglycemia, rash, liver toxicity) due to limited selectivity on ATP-binding site recognition in AKT proteins versus other protein kinases [7,10,21]. To overcome these toxicities, allosteric AKT inhibitors such as MK-2206 or our compound BAY 1125976 were developed. Indeed, MK-2206 showed a significantly reduced rate of Grade 3 hyperglycemia (3.0–9.3%) compared to the ATP-competitive AZD5363 (up to 39.0%) [22]. Compared to MK-2206, BAY 1125976 induced Grade 3 hyperglycemia only in one patient and also had only one case of Grade 3 rash in a patient from the 60 mg BID dose escalation cohort, indicating a more favorable tolerability, which might be due to the shorter half-life of BAY 1125976. Despite the strong pharmacodynamic coverage as evidenced by observed biomarker modulation, BAY 1125976 was able to induce a partial response in only one patient and stable disease in seven out of 28 patients treated in the 60 mg BID expansion cohort. The disease control rate was 28.6%. Interestingly, patients harboring the *AKT1^E17K^* mutation did not show signs of improved response over the general study population with no partial responses and only three out of nine patients harboring the mutation with stable disease, accounting for a disease control rate of 33.3%. Of the 14 patients with additional sequencing data available, six patients (all with PD as the best response) had an additional *PIK3CA* mutation, while other patients had mutations of FGFR signaling or cell cycle control genes such as *CDKN2A* (p16INK4) that could counteract the effects of AKT inhibition by BAY 1125976.

Our results are in line with those from other AKT inhibitors used in monotherapy in clinical trials [7]. The pan AKT inhibitor AZD5363 showed a similar pattern of target modulation with about 60% reduction in p-PRAS40 at 4 h and return to baseline within 24 h post-dosing and only limited evidence of antitumor activity in a breast cancer expansion cohort as well [23]. The allosteric AKT inhibitor MK-2206 showed only limited monotherapy activity against nasopharyngeal cancer [24], biliary tract cancer [25], or gastric cancer [26]. For MK-2206, which has a long half-life of 60–80 h, different dosing schedules were investigated, too. Both continuous QD and intermittent once weekly dosing demonstrated a robust inhibition of p-AKT. While the once weekly schedule significantly reduced treatment related side effects, it was not associated with sufficient clinical responses [27,28].

In our study, we were able to identify nine patients with the activating *AKT1^E17K^* point mutation based on prior investigator knowledge. Although preclinical data indicate a high sensitivity of *AKT1^E17K^* mutated tumor models to BAY 1125976 [10], these patients did not show signs of increased efficacy compared to patients with *AKT1* wild-type cancer. This is in contrast with the results from patients with *AKT1^E17K^* alterations receiving AZD5363, who showed longer progression-free survival [29]. Additional genomic analyses in our study revealed that most patients harbored additional oncogenic driver mutations. That means that *AKT1^E17K^* is not mutually exclusive to other alterations in the PI3K signaling pathway, which is in line with recent findings on the genomic characterization of metastatic HR+ breast cancer [23]. Higher sensitivity toward MK-2206 was hypothesized to be linked to additional alterations in the PI3K pathway, e.g., the loss of *PTEN* or mutation or amplification of *PIK3CA* [24,30]. Similar results were also reported for AZD5363 [29], although exclusive *PIK3CA* mutations did not correlate with increased response in another study in breast or gynecological cancer patients where concurrent mutations in *KRAS*, *NRAS*, *HRAS*, or *BRAF* were excluded [23]. Interestingly, patients with an allelic imbalance in *AKT1^E17K^* showed longer progression-free survival (PFS), while patients with subclonal mutations showed rapid disease progression in this study [29]. In our study, the best response was observed in an *AKT1* wild-type breast cancer patient who had an upstream *PIK3CA* mutation, which is in line with the finding of coincident *PI3KCA* mutations and improved PFS from another study [29]. In this study, patients with hotspot mutations in the ligand-binding domain of estrogen receptor α (*ESR1*) showed a shorter PFS. Next-generation sequencing (NGS) data from our study revealed that eight out of 14 evaluable patients also had mutations in *ESR1*, which could explain the overall disappointing efficacy.

Although overall the number of cases is small, data from our study and from the literature indicate that selecting patients based on *AKT1^E17K^* mutation alone is not sufficient to identify potential responders to such treatment. In a direct comparison, MK-2206 proved to be inferior to everolimus in renal cell cancer [31]. Combination approaches that include PI3K pathway inhibitors such as everolimus [32], buparlisib [33], or alpelisib [13,34,35] have shown promising results also in breast cancer patients, and additional preclinical data also confirm the combination potential for AKT inhibitors [36]. While a recent phase 2 study using MK-2206 and anastrozole in *PIK3CA*-mutant ER-positive breast cancer did not show additive efficacy [37], alpelisib in combination with fulvestrant received FDA approval for the treatment of patients with *PIK3CA*-mutated HR+ advanced breast cancer after previous endocrine therapy (PFS 11.0 versus 5.7 months) and also showed a treatment benefit in patients without *PIK3CA* mutation compared to placebo control (overall response rate 26.6% versus 12.8%) [38].

## 4. Materials and Methods

The study was approved by the respective independent ethics committees and institutional review boards for each study site (NCT Heidelberg: AFmu-327/2013; Kantonsspital St. Gallen: EKSG 13/064 / SG 355/13; Institut Gustave Roussy: 2012-004671-39 CPP 3099; MD Anderson Cancer Center: 2013-0727; Washington University School of Medicine: 201311119; UCLA: 13-001234), and it was conducted in accordance with the Declaration of Helsinki and Good Clinical Practice. All patients provided written informed consent before study participation.

### 4.1. Study Design

The study was a multi-center, open-label, non-randomized, phase I dose escalation study in patients with advanced solid tumors (NCT01915576). BAY 1125976 was applied orally in 21-day cycles. The primary objective was to determine the safety, tolerability, pharmacokinetics, and MTD in either continuous once daily (QD) or continuous twice daily (BID) dosing. Secondary objectives included assessment of pharmacokinetics (food effect), pharmacodynamic parameters, and tumor response. Furthermore, one of the objectives was to explore whether patients with tumor *AKT1^E17K^* mutation show an improved clinical benefit rate (partial response [PR] + stable disease [SD]) compared to patients without this tumor mutation. The study consisted of five dose escalation cohorts in the QD schedule and three dose escalation cohorts in the BID schedule. The expansion cohort at the BID MTD aimed to enrich for at least eight patients with an *AKT1^E17K^* mutation in a population of advanced, metastatic, or refractory solid tumors or HR+ metastatic breast cancer (Figure 1).

### 4.2. Treatment

BAY 1125976 was administered orally at a starting dose of 10 mg QD as a water-based liquid service formulation (LSF; 5 mg/mL), then changing to a tablet formulation at a dose of 40 mg QD. A bridging cohort compared the relative bioavailability of these two formulations. Dose escalation steps were 10 mg, 20 mg, 40 mg, 80 mg, and 120 mg QD. The BID dose escalation was initiated at 40 mg BID using the tablet formulation after the completion of the QD schedule and continued with 60 mg and 80 mg BID. The relative bioavailability of LSF versus the tablet was assessed on cycle 1, day −3 (3 days before the start of cycle 1) in the 20 mg and the 40 mg QD cohort. The effect of a high-fat meal on the PK of BAY 1125976 was assessed at the QD MTD after the administration of BAY 1125976 tablets immediately following consumption of a high-fat, high-calorie meal on cycle 1, day −3.

A dose-limiting toxicity (DLT) was defined as the occurrence of any of the following attributed to BAY 1125976 during cycle 1 of each dose level: absolute neutrophil count <0.5 × 109/L for ≥7 days; febrile neutropenia with absolute neutrophil count <0.5 × 109/L and a single body temperature >38.3 °C or a sustained temperature ≥38.0 °C for more than 1 h; platelets <25 × 109/L; persistent hematological toxicity ≥ Common Terminology Criteria for Adverse Events (CTCAE) Grade 3 for >7 days; any CTCAE Grade 3 or Grade 4 non-hematological toxicity related to study drug (excluding alopecia, nausea, and vomiting, not refractory to anti-emetics).

Dose escalation would not continue if the MTD had been defined with good precision according to model-based predictions using the continuous reassessment method or if the selected dose level for the next cohort had already been given in nine patients. Patients were treated until disease progression, unacceptable toxicity, consent withdrawal, or withdrawal from the study at the discretion of the investigator.

### 4.3. Inclusion Criteria

Patient were eligible if they were 18 years or older and had any kind of advanced, metastatic, or refractory solid tumors not amenable for standard therapy for the dose escalation parts and had histologically or cytologically proven HR+ metastatic breast cancer that was refractory to standard therapy and not amenable to curative surgery for the expansion part. At least eight patients with proven *AKT1^E17K^* mutation were planned to be included in the expansion part. Patients had to have evaluable and measurable disease according to the Response Evaluation Criteria in Solid Tumors (RECIST) version 1.1 and an Eastern Cooperative Oncology Group (ECOG) performance status of 0 to 2. Adequate bone marrow, liver, and renal functions were assessed by laboratory methods within 7 days before starting study treatment. All patients had to provide a tumor biopsy (either a new on study biopsy or an archival sample not older than 6 months) before treatment start. A second (paired) on-treatment biopsy on cycle 2, day 1 was mandatory for patients in the MTD cohort of the QD schedule for further pharmacodynamic biomarker analyses.

### 4.4. Safety, Response, and Pharmacokinetics Assessments

Safety evaluations included adverse events (AEs), vital signs, laboratory tests, and 12-lead electrocardiograms at screening, at planned times during each cycle, during follow-up, and up to 30 days after the last dose. Adverse events were graded using the National Cancer Institute Common CTCAE version 4.03. All patients receiving at least one dose of BAY 1125976 were included in the safety analysis set.

Investigators assessed tumor response according to RECIST v1.1 every two cycles. Pharmacokinetic assessments of single-dose and multiple-dose BAY 1125976 were planned in all patients in the dose escalation arms (single-dose in QD only), and in at least six patients at the MTD. Data were calculated using non-compartmental methods to estimate maximum drug concentration (C_max_) and area under the concentration time curve (AUC). The relative bioavailability of LSF and tablet were compared in the 20 and 40 mg cohorts of the QD dose escalation. The effect of food on compound exposure was compared in the 80 mg cohort.

### 4.5. Biomarker Assessments

A tumor biopsy was mandatory at screening (freshly taken or not older than six months) for all subjects. A pre-screening biopsy was mandatory for patients enrolled in the MTD expansion part of the study unless the *AKT1^E17K^* mutation status was confirmed previously. Otherwise, *AKT1^E17K^* mutations were investigated from fresh or archival biopsy samples using the Qiagen Therascreen AKT1 RGQ PCR Kit (Qiagen, Hilden, Germany) according to the manufacturer’s instructions. From new study biopsies, additional mutational profiling was performed by next-generation sequencing using FoundationOne^®^ T5a panel (Foundation Medicine, Cambridge, MA, USA).

Blood-based biomarker analyses were measured in platelet-rich plasma (PRP) to assess changes in the pharmacodynamics of the AKT pathway as phospho-AKT (p-AKT), total AKT, phosphor-PRAS40 (p-PRAS40) and total PRAS40. Briefly, 10 mL blood was collected with a S-Monovette^®^ Citrate (Sarstedt, Nümbrecht, Germany) using a 21G needle. After inverting the tube 8–10 times, samples were centrifuged at 400 g at room temperature for 4 min without brake. The upper PRP phase was separated into two vials and thrombin receptor-activating peptide (TRAP) or a control solution was added followed by incubation at 37 °C for 10 min in a thermoblock without shaking. After centrifugation of both samples at 800 g and +4 °C for 3 min, supernatant was discarded, and cells were lysed by adding completed lysis-buffer before cells were frozen at −70 °C in a precooled CoolSafe Chamber. Batched analysis using Mesoscale Discovery multiplex assays was performed (Nuvisan, Ulm, Germany).

### 4.6. PK/PD analysis

The pharmacokinetic/pharmacodynamic link between BAY 1125976 and the biomarkers p-AKT and p-PRAS40 was observed to show no evidence of hysteresis, and therefore was examined using nonlinear regression between plasma concentration and corresponding biomarker response in GraphPad Prism 7 (GraphPad Software Inc., La Jolla, CA, USA).

### 4.7. Statistical Analysis

Descriptive statistics were used to summarize demographic data, other baseline characteristics, AEs, safety parameters, and tumor assessment data. All subjects who received at least one dose of BAY 1125976 were included in the safety evaluation. All subjects who completed Cycle 1 with at least 80% of the required BAY 1125976 dose or discontinued during Cycle 1 due to a DLT were included in the MTD evaluation. All subjects who received at least one dose of BAY 1125976 and who have post-baseline efficacy data available were included in the efficacy evaluations. All subjects who completed the study were included in the evaluation of PD/PK. The incidence of subjects with DLTs during Cycle 1 was summarized by treatment and dose and modeled as a function of BAY 1125976 dose using Bayesian logistic regression. The incidence of treatment-emergent adverse events and drug-related adverse events, respectively, was summarized by treatment in frequency tables using the CTCAE grade.

## 5. Conclusions

In summary, our findings and data from the literature show that deeper genetic analyses and a thorough understanding of the underlying biological dependencies are necessary to clearly identify further pathway alterations that may positively or negatively impact on sensitivity toward AKT1 inhibition in monotherapy [7,39]. Taken holistically, these data suggest that AKT1 inhibition may remain an attractive target; however, an overarching appreciation for the presence of other oncogenic drivers and rigorous patient selection is likely necessary to markedly increase the probability of AKT1 inhibition becoming associated with robust efficacy.

## Figures and Tables

**Table 1:** Baseline patient demographics in the BAY 1125976 phase 1 study. BID: twice daily, ECOG: Eastern Cooperative Oncology Group, LSF: liquid service formulation, QD: continuous once daily, WT: wild-type.

Parameter	10 mg LSF QD	20 mg LSF QD	40 mg Tablet QD	80 mg Tablet QD	120 mg Tablet QD	40 mg Tablet BID	60 mg Tablet BID	80 mg Tablet BID	60 mg Expansion		Total
									Tablet (BID)		
									WT	*AKT1^E17K^*	
N	3	3	4	13	6	3	15	4	19	9	79
Mean age, years (range)	53.3 (44–67)	49.7 (37–64)	59.0 (47–75)	61.2 (48–75)	53.5 (37–69)	66.3 (50–82)	55.1 (40–74)	56.3 (38–70)	55.3 (31–76)	57.3 (41–73)	56.7 (31–82)
Females, n (%)	2 (66.7)	2 (66.7)	2 (50.0)	10 (76.9)	4 (66.7)	2 (66.7)	8 (53.3)	3 (75.0)	19 (100.0)	9 (100.0)	61 (77.2)
ECOG performance status, n (%)											
0	0	1 (33.3)	2 (50.0)	7 (53.8)	2 (33.3)	2 (66.7)	10 (66.7)	2 (50.0)	14 (73.7)	7 (77.8)	47 (59.5)
1	3 (100.0)	2 (66.7)	2 (50.0)	5 (38.5)	4 (66.7)	1 (33.3)	5 (33.3)	1 (25.0)	5 (26.3)	2 (22.2)	30 (38.0)
2	0	0	0	1 (7.7)	0	0	0	1 (25.0)	0	0	2 (2.5)
Prior systemic anticancer therapy, n (%)	3 (100.0)	3 (100.0)	4 (100.0)	13 (100.0)	6 (100.0)	2 (66.7)	15 (100.0)	4 (100.0)	19 (100.0)	9 (100.0)	78 (98.7)
Prior radiotherapy, n (%)	3 (100.0)	3 (100.0)	2 (50.0)	8 (61.5)	5 (83.3)	2 (66.7)	12 (80.0)	1 (25.0)	18 (94.7)	6 (66.7)	60 (75.9)

Patients with *AKT1^E17K^* mutation are also part of the 60 mg BID expansion cohort.

**Table 2:** BAY 1125976 related treatment-emergent adverse events occurring in ≥3 patients (Grade 3) or in ≥1 patient (Grade 4).

Adverse Events	10 mg LSF QD	20 mg LSF QD	40 mg Tablet QD	80 mg Tablet QD	120 mg Tablet QD	40 mg Tablet BID	60 mg Tablet BID	80 mg Tablet BID	60 mg Expansion Tablet (BID)		Total
									WT	*AKT1^E17K^*	
N	3	3	4	13	6	3	15	4	19	9	79
All, n (%)											
Grade 3	-	-	1 (25.0)	5 (38.5)	4 (66.7)	2 (66.7)	6 (40.0)	4 (100.0)	12 (61.1)	8 (88.9)	42 (53.2)
Grade 4	-	-	-	-	1 (16.7)	-	-	-	-	-	1 (1.3)
ALT increased, n (%)											
Grade 3	-	-	-	2 (15.4)	2 (33.3)	1 (33.3)	2 (13.3)	3 (75.0)	4 (21.0)	3 (33.3)	17 (21.5)
AST increased, n (%)											
Grade 3	-	-	-	2 (15.4)	3 (50.0)	-	3 (20.0)	2 (50.0)	7 (36.8)	3 (33.3)	20 (25.3)
AP increased, n (%)											
Grade 3	-	-	1 (25.0)	3 (23.1)	2 (33.3)	-	3 (20.0)	3 (75.0)	11 (57.9)	4 (44.4)	27 (34.2)
γ-GT increased, n (%)											
Grade 3	-	-	-	-	-	-	1 (6.7)	-	-	-	1 (1.3)
Grade 4	-	-	-	-	1 (16.7)	-	-	-	-	-	1 (1.3)

Patients with *AKT1^E17K^* mutation are also part of the 60 mg BID expansion cohort.

## Supplementary Materials

The following are available online at https://www.mdpi.com/2072-6694/11/12/1987/s1, Figure S1. Relative bioavailability of liquid vs. tablet formulation and food effect assessment, Figure S2. Full list of genetic aberrations found by NGS in samples from the breast cancer expansion cohort, Table S1. Baseline characteristics of patients—site of disease, Table S2. Non-compartmental pharmacokinetic parameters of BAY 1125976 after single and multiple dosing, Table S3. Relative bioavailability, Table S4. Food effect.
