# Supplementary material for: Phase 1 Dose Escalation Study of the Allosteric AKT Inhibitor BAY 1125976 in Advanced Solid Cancer—Lack of Association between Activating AKT Mutation and AKT Inhibition-Derived Efficacy
Source: Cancers (Basel). 2019 Dec 10;11(12):1987. doi: 10.3390/cancers11121987 (PMC6966663; doi:10.3390/cancers11121987)
Supplement: Supplementary file 1 [file cancers-11-01987-s001.pdf]

Table S1. Baseline characteristics of patients—site of disease.

Site of disease<br>N (%)	10 mg LSF QD<br>3 (100%)	20 mg LSF QD<br>3 (100%)	40 mg Tablet QD<br>4 (100%)	80 mg Tablet QD<br>13 (100%)	120 mg Tablet QD<br>6 (100%)	40 mg Tablet BID<br>3 (100%)	60 mg Tablet BID<br>15 (100%)	80 mg Tablet BID<br>4 (100%)	60 mg Expasion Tablet BID<br>28 (100%)	Total<br>79 (100%)
Abdomen	0	0	0	1 (7.7%)	1 (16.7%)	0	0	0	0	2 (2.5%)
Bone	0	1 (33.3%)	1 (25.0%)	5 (38.5%)	1 (16.7%)	0	3 (20.0%)	1 (25.0%)	20 (71.4%)	32 (40.5%)
Breast	0	0	0	0	0	0	0	0	1 (3.6%)	1 (1.3%)
Hilar lymph node	1 (33.3%)	2 (66.7%)	0	0	0	0	0	0	0	3 (3.8%)
Internal iliac lymph node	0	0	1 (25.0%)	0	0	0	0	0	0	1 (1.3%)
Kidney	0	0	0	0	1 (16.7%)	0	0	0	0	1 (1.3%)
Liver	0	1 (33.3%)	1 (25.0%)	6 (46.2%)	2 (33.3%)	0	6 (40.0%)	2 (50.0%)	26 (92.9%)	44 (55.7%)
Liver hilum lymph node	0	0	0	0	0	0	1 (6.7%)	0	0	1 (1.3%)
Mediastinal lymph node	1 (33.3%)	0	0	1 (7.7%)	0	0	0	0	0	2 (2.5%)
Other	3 (100.0%)	3 (100.0%)	4 (100.0%)	10 (76.9%)	4 (66.7%)	3 (100.0%)	12 (80.0%)	3 (75.0%)	17 (60.7%)	59 (74.7%)
Para-aortic lymph node	0	1 (33.3%)	0	1 (7.7%)	0	0	2 (13.3%)	0	0	4 (5.1%)
Parasternal lymph node	0	0	0	0	0	0	1 (6.7%)	0	0	1 (1.3%)
Paratracheal lymph node	0	0	0	0	1 (16.7%)	0	0	0	0	1 (1.3%)
Portal lymph node	0	0	1 (25.0%)	0	0	0	1 (6.7%)	0	1 (3.6%)	3 (3.8%)
Retroperitoneum	0	0	1 (25.0%)	1 (7.7%)	0	0	0	1 (25.0%)	0	3 (3.8%)

Table S2. Non-compartmental pharmacokinetic parameters of BAY 1125976 after single and multiple dosing.

Parameter	Dose, Formulation, Schedule								
	**10 mg LSF QD**	**20 mg LSF QD**	**40 mg Tablet QD**	**80 mg Tablet QD**	**120 mg Tablet QD**	**40 mg Tablet BID**	**60 mg Tablet BID**	**80 mg Tablet BID**	**60 mg Expansion Tablet BID**
N	3	3	4	13	6	3	15	4	9
Cycle 1, Day 1									
C_{max} (µg/L)	2.65 / 66.5<br>(1.36–4.44)	8.00 / 32.0<br>6.45–11.4)	8.65 / 65.1<br>(3.76–13.5)	38.5 / 49.9<br>(15.7–78.6)	36.9 / 47.2<br>(17.6–69.3)	13.5 / 32.1<br>(9.4–16.6)	23.1 / 72.9<br>(7.51–58.1)	26.0 / 24.4<br>(20.6–36.4)	17.3 / 85.9<br>(5.83–52.7)
AUC (µg * h/L)	−/−<br>(28.5–30.3)[a]	95.4 / 93.2<br>(59.1–238)	74.0 / 8.76<br>(67.0–79.1)[b]	289 / 54.9<br>(98.4–739)	235 / 42.7<br>(122–322)	−	−	−	−
t_{max} (h) [d]	1.0<br>(0.5–3.9)	1.9<br>(1.1–2.0)	1.8<br>(1.0–8.0)	1.0<br>(0.5–4.1)	1.3<br>(1.0–2.1)	1.0<br>(1.0–3.0)	2.0<br>(0.5–4.0)	3.0<br>(0.8–4.0)	2.0<br>(1.0–4.0)
Cycle 2, Day 1									
C_{max} (µg/L)	2.17 / 50.0<br>(1.26–3.04)	−/−<br>(6.76–9.48)[a]	9.21 / 69.6<br>(3.99–16.5)	37.7 / 87.1<br>(13.8–123)[c]	44.5 / 26.8<br>(36.1–59.9)[b]	18.0 / 36.5<br>(12.0–23.3)	33.4 / 101<br>(10.0–131)[e]	−/−<br>(32.4–84.2)[a]	40.4 / 73.9<br>(18.9–84.6)[g]
AUC (µg * h/L) [f]	13.4 / 43.6<br>(8.89–20.5)	−/−<br>(65.7–106)[a]	127 / 19.2<br>(106–156)	363 / 93.0<br>(94.5–1010)[c]	294 / 19.9<br>(240–356)[b]	94.0 / 22.4<br>(81.6–121)	177 / 80.7<br>(76.5–824)[e]	−/−<br>(186–387)[a]	195 / 44.0<br>(111–347)[g]
t_{max} (h) [d]	0.6<br>(0.5–1.0)	−<br>(1.1–1.9)[a]	2.1<br>(1.0–4.1)	2.0<br>(1.0–4.1)[c]	2.0<br>(1.5–2.0)[b]	1.0<br>(1.0–2.0)	2.0<br>(1.0–4.1)[e]	−<br>(1.0–1.0)[a]	1.5<br>(0.5–4.2)[g]

The pharmacokinetics analysis set consisted of 60 patients. Shown is the geometric mean, % coefficient of variation and range for single (Cycle 1, Day 1) and multiple (Cycle 2, Day 1) dosing of BAY 1125976. Single dose PK was assessed in the QD schedule only. [a] n = 2; [b] n = 3; [c] n = 8; [d] median (range); [e] n = 11; [f] shown is AUC (0-t_{last}); [g] n = 6.

Table S3. Relative bioavailability. Comparison of a liquid versus solid formulation of BAY 1125976 at dose levels 20 mg and 40 mg QD (geometric mean (%CV)).

Parameter	Dose, Formulation, Schedule			
	20 mg QD		40 mg QD	
	LSF (C1D1); *n* = 3	Tablet (C1D3); *n* = 3	LSF (C1D1); *n* = 4	Tablet (C1D3); *n* = 4
C_{max}/D* (1/L * 10^{-3})	0.372 (36.4)	0.456 (39.1)	0.207 (62.7)	0.216 (65.1)
AUC(0-48)/D* (h/L * 10^{-3})	4.23 (91.0)	3.91 (75.0)	1.88 (18.2)	1.80 (8.30)[b]
t_{max} [a] (h)	1.9 (1.1-2.0)	1.1 (1.0-2.0)	2.5 (1.0-4.2)	1.8 (1.0-8.0)

C: cycle, D: day; [a] median (range); [b] *n* = 3.

Table S4. Food effect. Pharmacokinetic parameters of BAY 1125976 in plasma after single dose of BAY 1125976 given in fasted vs. fed condition (geometric mean (%CV)).

Parameter	80 mg QD	
	Fed condition (C1D-3); *n* = 3	Fasted condition (C1D1); *n* = 3
C_{max} (μg/L)	21.1 (36.6)	28.8 (64.9)
AUC(0-48) (μg * h/L)	192 (52.7)	165 (52.4)
t_{max} [a](h)	4.1 (1.0-4.1)	1.0 (0.5-2.1)

C: cycle, D: day; [a] median (range)

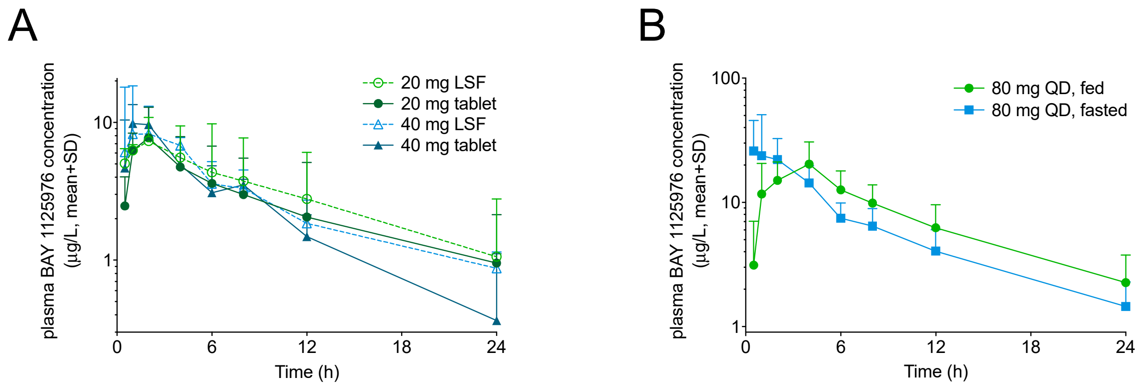

Figure S1. (**A**) Relative bioavailability of liquid vs. tablet formulation showing bioequivalence at two tested dose levels. (**B**) Food effect assessment at 80 mg QD.

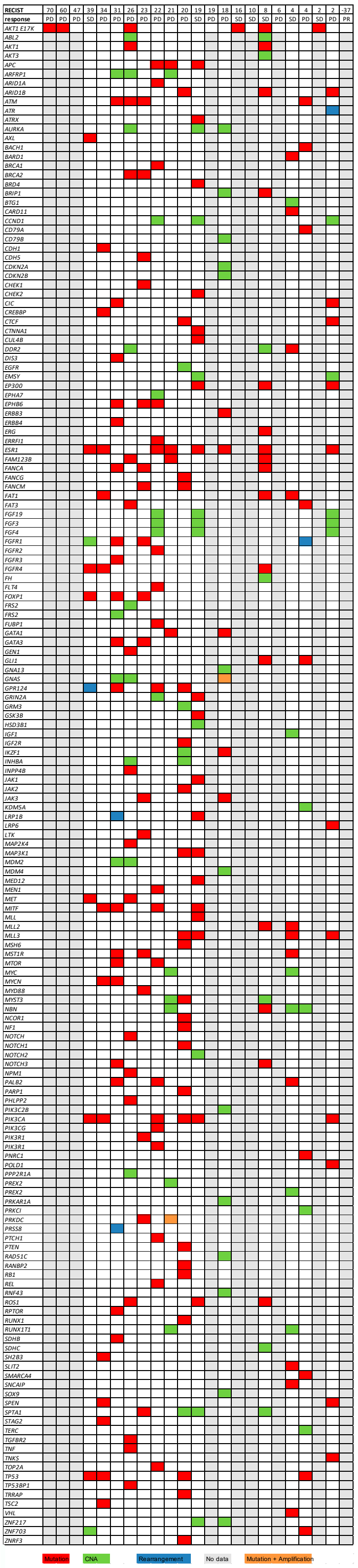

Figure S2. Full list of genetic aberrations found by NGS in samples from the breast cancer expansion cohort. Shown is the response assessment according to RECIST v1.1 as progressive disease (PD), stable disease (SD) or partial response (PR) and the best change of tumor size as & change from baseline. Grey: no data available (failed analysis; low/no tumor content in sample; missing sample). Red: mutation. Yellow: copy number alteration (amplification). Blue: rearrangement. Orange: mutation and amplification.
